# Commentary: Harmane potentiates nicotine reinforcement through MAO-A inhibition at the dose related to cigarette smoking

**DOI:** 10.3389/fnmol.2023.1119538

**Published:** 2023-02-07

**Authors:** Penelope Truman

**Affiliations:** School of Health Sciences, College of Health, Massey University, Wellington, New Zealand

**Keywords:** tobacco smoking, monoamine oxidase inhibitors, harmane, tobacco dependence, nicotine

This paper (Ding et al., 2022) compares the behavioral and biochemical effects of nicotine and nicotine supplemented with harmane (also called harman) a well-known monoamine oxidase (MAO) inhibitor found in tobacco smoke.

In this paper, the case for harmane potentiating nicotine's behavioral effects is well made, but I wonder if there has not been a miscommunication around the author's claim that the concentration of harmane used equates to that found in tobacco smoke. The concentration ratio of harmane: nicotine used was 1:30. Other literature, where harmane and nicotine have been directly measured in tobacco smoke, suggests a range of 0.1 to 6 micrograms per cigarette, compared to milligrams of nicotine per cigarette—giving a ratio of more like 1:1,000 (Herraiz, 2004; Talhout et al., 2007; Rodgman and Perfetti, 2013; Truman et al., 2017).

To explain how they reached the 1:30 ratio used in their study, Ding et al. refer to Jaccard et al. (2019), who measured nicotine in reference cigarettes and van der Toorn et al. (2019), who measured the total amount of MAO inhibitory activity in reference cigarettes compared to that in heated tobacco products. Nowhere in these two papers is a direct measure of harmane's concentration in cigarette smoke. I can only suggest that, in the current paper, the amount of harmane was adjusted to match the total amount of MAO-A inhibitory activity in tobacco smoke. Further, the Ding et al. results for the 0.1 H condition (which showed only marginal behavioral effects) is largely in line with results from other workers (Smith et al., 2015) who assessed the effect of harman (co-administered with other potentially relevant tobacco compounds, including acetaldehyde) on nicotine self-administration, at a harman: nicotine ratio of 1:300.

In a 2017 paper (Truman et al., 2017) we determined a harman: nicotine ratio of close to 1:1,000 for one tobacco type, and 1:300 for norharman: nicotine, in agreement with earlier work (Herraiz, 2004; Talhout et al., 2007; Rodgman and Perfetti, 2013). We showed that the amount of harman plus norharman found in this tobacco smoke provided less than 1/10th of the total MAO-A inhibitory activity of the tobacco smoke.

Thus, Ding and co-workers appear to have given a demonstration of the effect on nicotine self-administration of adding harmane in an amount sufficient to replicate the total MAO-A inhibitory activity measured in tobacco smoke samples, which happens to be at least 10-fold higher than the actual amount of harmane in the same tobacco smoke samples.

This is a very useful paper since it shows that, at least in rats, the amount of MAO inhibition that a smoker would encounter when smoking enhances the dopamine response to nicotine and makes the inhaled nicotine more reinforcing than it would be on its own. However, it should not be taken as indicating that, in smokers, harmane alone explains the monoamine oxidase inhibitory effects seen in smokers. Tobacco smoke contains a wide variety of MAO inhibitors (Hong et al., 2022) but the overall importance of these, individually, is as yet unclear.

## Author contributions

The author confirms being the sole contributor of this work and has approved it for publication.
